# Predictive value of changes in temporal muscle volume for 6-month functional outcome in patients with hypertensive intracerebral hemorrhage

**DOI:** 10.3389/fneur.2026.1751620

**Published:** 2026-08-12

**Authors:** Hongzuo Lv, Yibo Xin, Guihan Zhang, Wenbo Liu, Jinsheng Ye, Zhenyu Li, Dahai Zheng

**Affiliations:** Department of Neurosurgery, The Eighth Affiliated Hospital, Southern Medical University (The First People’s Hospital of Shunde, Foshan), Foshan, China

**Keywords:** computed tomography, hypertensive intracerebral hemorrhage, prognostic prediction, sarcopenia, temporal muscle volume

## Abstract

**Background and purpose:**

Accurate prediction of functional outcome after hypertensive intracerebral hemorrhage (HICH) remains challenging. Temporal muscle volume (TMV), measurable on routine head computed tomography (CT), may reflect skeletal muscle status. This study investigated longitudinal TMV changes, the factors associated with these changes, and their prognostic value in HICH.

**Materials and methods:**

This single-center retrospective study included 149 patients with HICH involving the thalamus or basal ganglia admitted between January 2022 and December 2023. TMV was measured on admission and follow-up CT scans during weeks 1–3. Absolute temporal muscle volume loss (ATMVL) and temporal muscle volume reduction rate (TMVRR) were calculated relative to baseline. Six-month functional outcome was assessed using the modified Rankin Scale; scores of 3–6 indicated an unfavorable outcome.

**Results:**

Mean admission TMV was 32.3 ± 13.5 cm^3^. TMVRR and ATMVL increased significantly over time, averaging 4.63 percentage points and 1.65 cm^3^ per week, respectively. Surgical treatment and greater baseline TMV were independently associated with higher week 2 TMVRR and ATMVL. Patients with unfavorable outcome showed greater TMVRR at weeks 2 and 3 and greater ATMVL at week 2. After adjustment for age, sex, and hemorrhage volume, week 2 TMVRR (OR = 1.109, *p* = 0.004) and ATMVL (OR = 1.282, *p* = 0.009) were independently associated with an unfavorable 6-month outcome, whereas admission TMV was not.

**Conclusion:**

Temporal muscle loss progresses during hospitalization in patients with HICH. Week 2 TMVRR and ATMVL may provide practical CT-based markers that complement established predictors of functional outcome.

## 1 Introduction

Intracerebral hemorrhage (ICH) accounts for approximately 10–20% of all strokes and remains a major cause of mortality and long-term disability worldwide, with more than two-thirds of survivors experiencing functional dependence (1). Hypertensive intracerebral hemorrhage (HICH) is the most common type of ICH, often occurring in the thalamus and basal ganglia. Nevertheless, predicting long-term functional outcome in HICH patients remains challenging because reliable and readily accessible biomarkers are limited.

Stroke increases the risk of sarcopenia (2), which is independently associated with poor prognosis (3) and reduced rehabilitation effectiveness (4). Early identification of sarcopenia is therefore important in patients with stroke. According to the European Working Group on Sarcopenia in Older People (EWGSOP), low muscle strength is a key indicator of sarcopenia, and grip strength and walking tests are commonly used to evaluate muscle function (5). However, these assessments are often impractical in patients with HICH, particularly those admitted to neurocritical care units, because sedation, impaired consciousness, and neurological deficits may preclude active participation.

Muscle mass can be evaluated using total or appendicular skeletal muscle mass or the cross-sectional area of specific muscle groups (6). The area or volume of the psoas muscle at the third lumbar vertebral level on abdominal computed tomography (CT) has also been used to identify sarcopenia (7, 8). However, abdominal and limb imaging is not routinely performed in patients with HICH, and additional examinations may increase costs, prolong assessment, and expose critically ill patients to avoidable transfer-related risks. In contrast, head CT is routinely performed for the diagnosis and monitoring of HICH. Temporal muscle thickness (TMT) measured on head CT has therefore been proposed as a practical surrogate marker of skeletal muscle mass (9). In neurocritical care patients, TMT progressively decreases during hospitalization in parallel with rectus femoris muscle loss (10). Lower TMT has also been associated with dysphagia (11) and unfavorable outcome after stroke (12, 13). These findings suggest that temporal muscle assessment using routine head CT may enable simultaneous evaluation of neurological disease and muscle status without additional imaging.

In addition to TMT, temporal muscle area (TMA) (14, 15) and temporal muscle volume (TMV) (16) have emerged as alternative imaging-based measures. However, TMT and TMA are generally obtained from a single CT slice and may not fully represent the overall temporal muscle mass. In contrast, TMV above the zygomatic arch captures the three-dimensional volume of the temporal muscle and may be less affected by anatomical variation, head positioning, and slice selection, potentially improving measurement accuracy and reproducibility.

Previous studies of temporal muscle measurements in patients with stroke have mainly relied on CT images obtained at admission or during the early disease stage, with limited attention to muscle loss during hospitalization. Moreover, the clinical significance and prognostic value of longitudinal changes in TMV among patients with HICH remain unclear.

Therefore, this study aimed to characterize changes in TMV during hospitalization in patients with HICH and compare these changes according to disease severity and functional outcome; identify clinical factors associated with TMV loss; examine the relationships of baseline TMV and longitudinal TMV changes with clinical complications; and evaluate their value in predicting functional outcome.

## 2 Materials and methods

### 2.1 Study design and participants

This single-center retrospective study included 149 patients with HICH involving the thalamus or basal ganglia who were admitted to the Department of Neurosurgery, The Eighth Affiliated Hospital, Southern Medical University (The First People’s Hospital of Shunde, Foshan), between January 2022 and December 2023. The inclusion criteria were as follows: (1) diagnosis within 2 days of symptom onset; (2) age >18 years; and (3) availability of an initial non-contrast head computed tomography (CT) scan obtained at admission and at least one follow-up CT scan obtained on days 5–9(week 1), 12–16(week 2), or 19–23(week 3) after admission. The exclusion criteria were as follows: (1) insufficient CT image quality for TMV measurement or no follow-up CT scan within any of the predefined time windows; (2) discharge within 7 days or admission to the neurosurgical department more than 2 days after symptom onset; (3) major comorbidities, including malignancy or severe chronic renal, cardiac, or pulmonary disease; (4) severe in-hospital complications, including massive pericardial effusion, recurrent intracranial hemorrhage, or cerebral infarction; and (5) a pre-stroke modified Rankin Scale (mRS) score ≥2. The study protocol was approved by the Ethics Committee of The Eighth Affiliated Hospital, Southern Medical University (The First People’s Hospital of Shunde, Foshan) (approval number: KYLS20251206). Informed consent was obtained from all patients or their legally authorized representatives.

### 2.2 Patient treatment

All patients were managed in accordance with the Chinese guidelines for the diagnosis and treatment of HICH (17). Upon admission, patients were evaluated for coagulation disorders and hematological diseases. Cranial computed tomography angiography was performed to exclude underlying cerebrovascular abnormalities, including intracranial aneurysms, arteriovenous malformations, and moyamoya disease. Patients without indications for surgery received guideline-based conservative treatment. Patients with supratentorial hemorrhage who met the surgical criteria underwent hemorrhage evacuation, neuroendoscopic hemorrhage removal, minimally invasive puncture and drainage, or decompressive craniectomy, as clinically appropriate. External ventricular drainage was performed in patients with intraventricular hemorrhage when indicated.

### 2.3 Data collection

Baseline demographic, clinical, laboratory, and imaging data were collected from the electronic medical records. Hemorrhage volume was determined by manually segmenting the hemorrhage on admission CT images using 3D Slicer software. The median hemorrhage volume of the cohort was 19.2 mL and was used to categorize patients into a low-volume group (<19.2 mL) and a high-volume group (≥19.2 mL).

Functional outcome was assessed 6 months after HICH onset by telephone interview using the mRS. Assessments were conducted by a trained nurse who was blinded to the patients’ clinical and imaging data. When a patient could not be contacted directly or was unable to communicate, a family member or caregiver familiar with the patient’s daily functional status was interviewed as a proxy respondent. An mRS score of 0–2 was defined as a favorable functional outcome, whereas a score of 3–6 was defined as an unfavorable functional outcome. In-hospital infections were diagnosed according to the relevant Chinese national diagnostic and treatment guidelines.

### 2.4 Temporal muscle volume measurement

Because follow-up head CT examinations are commonly performed during the first 3 weeks after HICH, CT scans obtained during this period were used to evaluate longitudinal changes in TMV. Follow-up examinations performed on days 5–9, 12–16, and 19–23 after admission were designated as the week 1, week 2, and week 3 time points, respectively. When more than one eligible CT scan was available within a predefined time window, only one scan was included in the analysis. Given the substantial inter-individual variation in baseline TMV, both absolute TMV and changes relative to baseline were assessed. Absolute temporal muscle volume loss (ATMVL) and the temporal muscle volume reduction rate (TMVRR) were calculated as follows: ATMVL = TMV₀ − TMV₁, and TMVRR = (TMV₀ − TMV₁) / TMV₀ × 100% (where TMV_0_ represents TMV measured on the admission CT scan and TMVₜ represents TMV measured at the corresponding follow-up time point). For patients receiving conservative treatment, the mean TMV of the bilateral temporal muscles was used. For patients undergoing surgery, only the temporal muscle on the non-operative side was measured. If both temporal muscles were affected by surgery, the corresponding CT examination was excluded from the TMV analysis.

**Figure 1:**
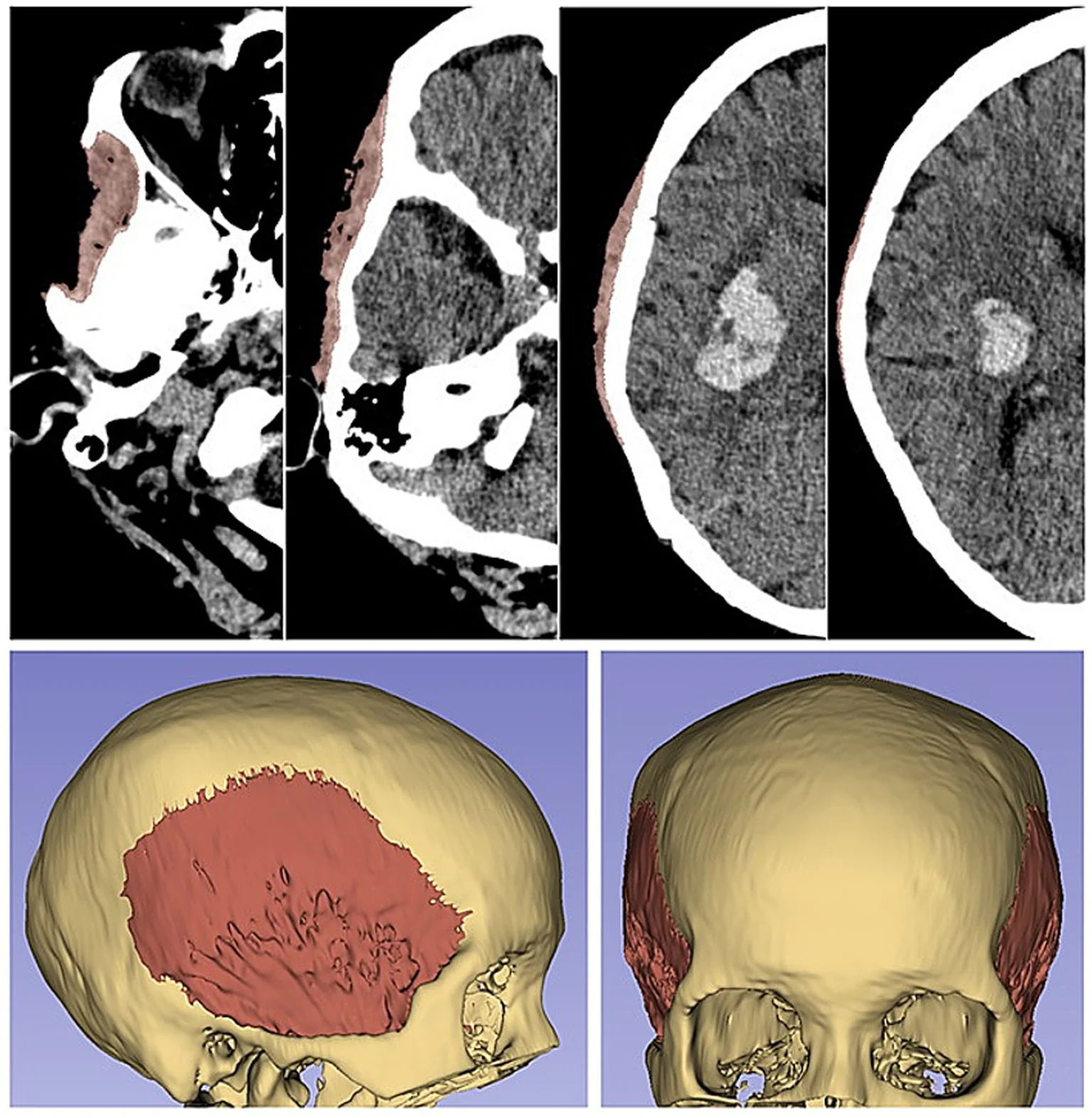
Representative CT images and three-dimensional reconstruction of the temporal muscles in a 62-year-old woman with hypertensive intracerebral hemorrhage. The hemorrhage was located in the right basal ganglia. The temporal muscles were manually segmented, as indicated by the red regions, and bilateral temporal muscle volumes were reconstructed and calculated using 3D Slicer software.

All TMV measurements were performed by a neurosurgeon who was blinded to the patients’ clinical information and the chronological order of the CT scans. Only CT images with a slice thickness of <1.5 mm were included. Digital Imaging and Communications in Medicine data were imported into 3D Slicer software (version 5.6.1). In the Segment Editor module, the temporal muscle was manually delineated using the Paint tool. The Scissors tool was then used to isolate the portion of the temporal muscle located between the zygomatic arch and the inferior temporal line, with the cranial bone forming the medial boundary and the temporal fascia forming the lateral boundary. Three-dimensional reconstruction was subsequently performed, and the volume of the segmented temporal muscle was automatically calculated using 3D Slicer, as illustrated in Figure 1. The admission hemorrhage was segmented separately using the same software, and its three-dimensional volume was calculated from the reconstructed region of interest.

### 2.5 Data presentation, and statistical analysis

Statistical analyses were performed using SPSS version 29.0 and R version 4.3.1. Categorical variables are presented as numbers and percentages. Normally distributed continuous variables are expressed as the mean ± standard deviation, whereas non-normally distributed variables are presented as the median and interquartile range. Differences in TMV between groups defined by patient characteristics were assessed using independent-samples t tests. One-way analysis of variance was used to compare TMV, ATMVL, and TMVRR across the predefined time points. Linear regression analyses were performed to identify clinical characteristics and complications associated with changes in TMV. Logistic regression analyses were used to evaluate the associations of TMV and its longitudinal changes with 6-month functional outcome. The discriminative performance of hemorrhage volume, the ICH Score, and TMV-related parameters for predicting 6-month functional outcome was evaluated using receiver operating characteristic (ROC) curve analysis. The areas under the ROC curves were compared using DeLong’s test. Owing to the retrospective design, not all patients underwent head CT examinations at every predefined follow-up time point. Therefore, analyses at each time point included all patients with a valid TMV measurement at that time point rather than being restricted to patients with complete measurements at all four time points. For logistic regression analyses evaluating the prognostic value of week 2 TMV changes, only patients with valid week 2 ATMVL and TMVRR measurements and available 6-month mRS data were included. All statistical tests were two-sided, and a *p* value <0.05 was considered statistically significant.

## 3 Results

Of the 648 consecutive patients with HICH who were screened, 457 did not meet the inclusion criteria. A further 33 patients were excluded because of insufficient CT image quality, five because of transfer to another department within 7 days of admission, and four because they were admitted more than 2 days after symptom onset. Ultimately, 149 patients were included in the final analysis.

The demographic and clinical characteristics, initial clinical presentation, treatment procedures, in-hospital complications, and 6-month functional outcome of the included patients are summarized in Table 1.

**Table 1:** Baseline characteristics and prognostic analysis of patients (*n* = 149).

Patient characteristics (*n* = 149)	n (%) or median (IQR)
Age (years)	56(48–65)
Female sex	46(31)
Diabetes	13(9)
Stroke history	15(10)
Smoking history	24(16)
Alcohol abuse history	16(11)
ICH volume	19(7–41)
GCS score	
13–15	68(45.5)
9–12	31(21)
3–8	50(33.5)
ICH score	
0	35(23.5)
1	44(30)
2	34(22.5)
3	28(19)
4	8(5)
5	0(0)
Intraventricular hemorrhage	82(55)
Treatment	
Conservative treatment	64(43)
Surgical treatment	85(57)
Pneumonia	87(58)
Intracranial infection	7(5)
Urinary tract infection	15(10)
Bloodstream infection	4(3)
Length of hospital stay (days)	25.5(18.5–30.5)
6-month mRS score	
0	14(9)
1	33(22)
2	22(15.5)
3	20(13.5)
4	21(14)
5	23(15)
6	9(6)
Lost to follow-up	7(5)

Data are presented as median (interquartile range) for continuous variables and as number (percentage) for categorical variables. ICH, intracerebral hemorrhage; GCS, Glasgow Coma Scale; mRS, modified Rankin Scale; IQR, interquartile range.

A total of 415 head CT examinations were analyzed, comprising 149 scans obtained at admission, 116 at week 1, 106 at week 2, and 44 at week 3. The mean baseline TMV was 32.3 ± 13.5 cm^3^. Patients aged ≥65 years had a significantly lower TMV than those aged <65 years, and women had a significantly lower TMV than men (both *p* < 0.001). TMV did not differ significantly according to diabetes status (*p* = 0.749), smoking history (*p* = 0.132), or history of alcohol abuse (*p* = 0.227).

### 3.1 Temporal muscle volume changes over time

**Figure 2:**
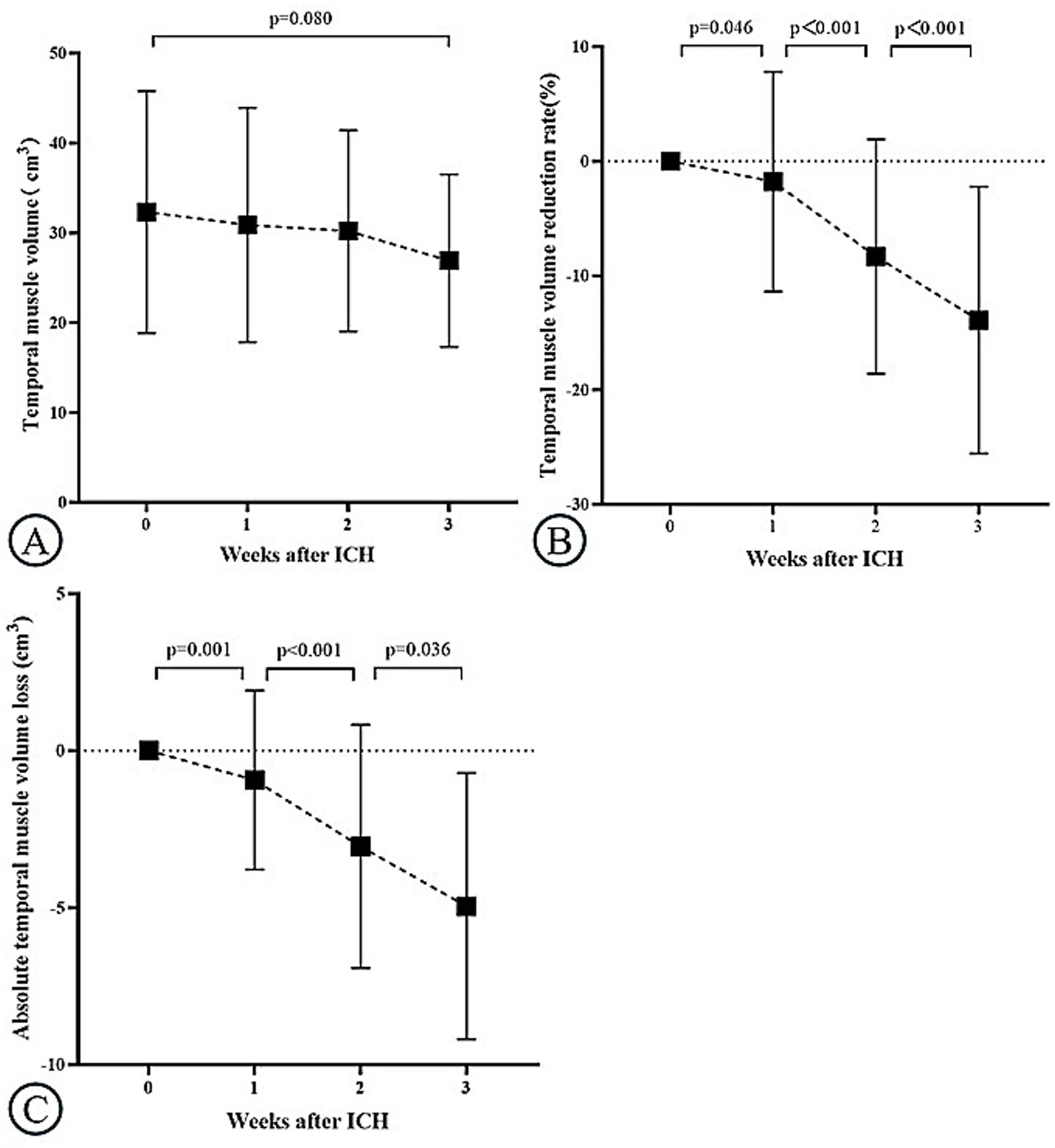
Longitudinal changes in temporal muscle volume during hospitalization in patients with HICH. **(A)** Temporal muscle volume (TMV); **(B)** Temporal muscle volume reduction rate (TMVRR); and **(C)** Absolute temporal muscle volume loss (ATMVL). Brackets above the plots indicate pairwise comparisons between different time points.

TMV decreased progressively during hospitalization, although the overall difference across time points did not reach statistical significance (*p* = 0.080; Figure 2A). In contrast, both TMVRR and ATMVL increased significantly over time (both *p* < 0.05). The mean TMVRR was 1.80% ± 9.61% at week 1, 8.33% ± 10.24% at week 2, and 13.90% ± 11.66% at week 3, corresponding to an average increase of approximately 4.63 percentage points per week (Figure 2B). Similarly, the mean ATMVL increased from 0.94 ± 2.85 cm^3^ at week 1 to 3.05 ± 3.87 cm^3^ at week 2 and 4.96 ± 4.23 cm^3^ at week 3, representing an average increase of approximately 1.65 cm^3^ per week (Figure 2C). These findings demonstrate progressive temporal muscle loss during hospitalization in patients with HICH.

**Figure 3:**
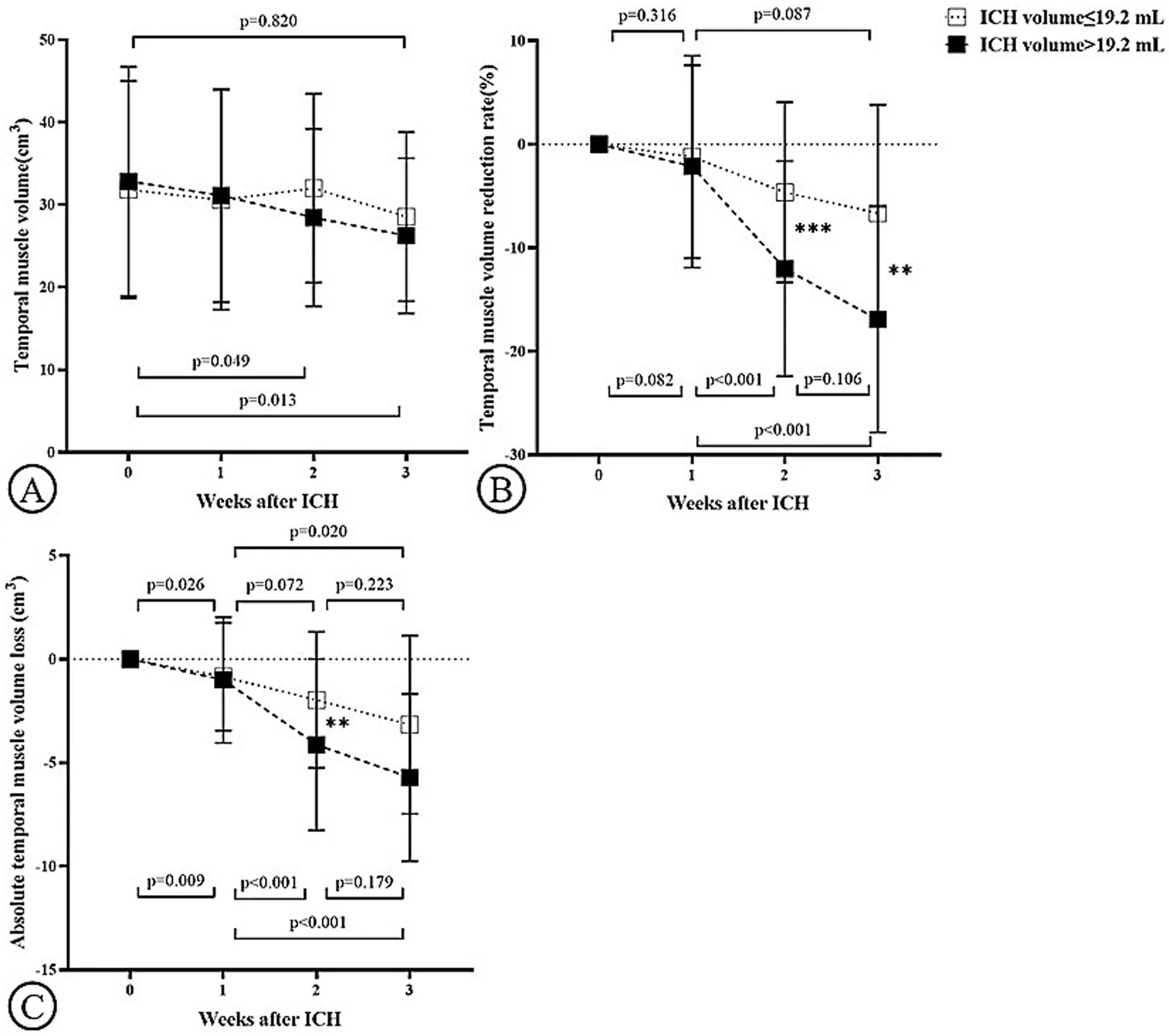
Longitudinal changes in temporal muscle parameters according to hemorrhage volume. Changes in **(A)** temporal muscle volume (TMV), **(B)** temporal muscle volume reduction rate (TMVRR), and **(C)** absolute temporal muscle volume loss (ATMVL) during hospitalization are shown for the low hemorrhage volume group (<19.2 mL; white squares) and the high hemorrhage volume group (≥ 19.2 mL; black squares). Brackets indicate pairwise comparisons between time points within each group or between the two hemorrhage volume groups at the corresponding time points. Asterisks indicate statistically significant between-group differences (* *p* < 0.05, ** *p <* 0.01, *** *p <* 0.001).

Patients were stratified into a low hemorrhage volume group (<19.2 mL) and a high hemorrhage volume group (≥19.2 mL) according to the cohort median hemorrhage volume of 19.2 mL. In the high-volume group, TMV decreased progressively over time (*p* = 0.049), with significant differences observed between baseline and week 2 (*p* = 0.049) and between baseline and week 3 (*p* = 0.013; Figure 3A). In parallel, both TMVRR and ATMVL increased over time in this group, with average increases of approximately 5.643 percentage points and 1.903 cm^3^ per week, respectively.

Between-group comparisons showed no significant difference in TMV at any time point (Figure 3A). However, TMVRR was significantly higher in the high-volume group than in the low-volume group at week 2 (12.018% ± 10.403% vs. 4.641% ± 8.711%, *p* < 0.001) and week 3 (16.932% ± 10.899% vs. 6.657% ± 10.481%, *p* = 0.006; Figure 3B). Similarly, ATMVL was significantly greater in the high-volume group at week 2 (4.135 ± 4.132 cm^3^ vs. 1.972 ± 3.286 cm^3^, *p* = 0.004; Figure 3C). These findings suggest that greater hemorrhage volume is associated with more pronounced temporal muscle loss during hospitalization (Table 2).

**Table 2:** Univariate linear regression analysis of factors affecting the reduction in temporal muscle volume.

Variable	TMVRR (%) at Week 2				ATMVL (cm^3^) at Week 2			
	β	95% CI	*p* value	R^2^	β	95% CI	*p* value	R^2^
Sex								
Male	Ref				Ref			
Female	−0.518	(−4.874, 3.839)	0.814	0.001	−1.450	(−3.072, 0.172)	0.079	0.029
Age (years)	−0.036	(−0.184, 0.111)	0.628	0.002	−0.055	(−0.109, 0.000)	**0.049**	0.037
Diabetes								
No	Ref				Ref			
Yes	3.479	(−5.072, 12.031)	0.422	0.006	0.740	(−2.498, 3.979)	0.651	0.002
Smoking history								
No	Ref				Ref			
Yes	−1.229	(−6.111, 3.653)	0.619	0.002	−0.410	(−2.256, 1.435)	0.660	0.002
Alcohol abuse								
No	Ref				Ref			
Yes	1.759	(−4.275, 7.792)	0.564	0.003	0.956	(−1.320, 3.233)	0.407	0.007
Hemorrhage Volume (mL)	0.112	(0.045, 0.180)	**0.001**	0.094	0.024	(−0.003, 0.051)	0.076	0.030
Intraventricular hemorrhage								
No	Ref				Ref			
Yes	5.279	(1.423, 9.135)	**0.008**	0.066	0.029	(−0.040, 0.098)	0.404	0.013
Midline Shift (mm)	0.526	(0.136, 0.915)	**0.009**	0.064	0.123	(−0.028, 0.273)	0.108	0.025
Treatment								
Conservative	Ref				Ref			
Surgical	8.916	(5.322, 12.510)	**<0.001**	0.189	3.066	(1.681, 4.451)	**<0.001**	0.156
ICH score								
0	Ref				Ref			
1	−1.035	(−4.081, 6.152)	**0.045**	0.144	0.099	(−1.909, 2.106)	0.923	0.077
2	7.682	(2.007, 13.356)	**0.008**		2.286	(0.060, 4.513)	**0.044**	
3	7.562	(1.958, 13.165)	**0.009**		2.225	(0.026, 4.423)	**0.047**	
4	11.531	(2.817, 20.246)	**0.010**		1.662	(−1.757, 5.082)	0.337	
GCS Score	−1.006	(−1.528, −0.484)	**<0.001**	0.123	−0.308	(−0.510, −0.106)	**0.003**	0.081
Baseline TMV (cm^3^)	0.171	(0.020, 0.322)	**0.027**	0.046	0.169	(0.121, 0.217)	**<0.001**	0.316
NLR	0.093	(−0.219, 0.405)	0.555	0.003	0.069	(−0.048, 0.186)	0.244	0.013
PLR	−0.010	(−0.030, 0.010)	0.312	0.010	−0.002	(−0.010, 0.005)	0.530	0.004
Total Protein (g/L)	−0.030	(−0.236, 0.176)	0.774	0.001	0.005	(−0.074, 0.084)	0.892	0.000
Albumin (g/L)	−0.254	(−0.748, 0.241)	0.311	0.010	−0.031	(−0.222, 0.159)	0.744	0.001
Pulmonary infection								
No	Ref				Ref			
Yes	4.718	(0.771, 8.666)	**0.020**	0.051	0.884	(−0.638, 2.406)	0.252	0.013
Urinary tract infection								
No	Ref				Ref			
Yes	0.525	(−6.000, 7.040)	0.873	0.000	0.232	(−2.234, 2.261)	0.855	0.000
Intracranial infection								
No	Ref				Ref			
Yes	−0.696	(−9.273, 7.881)	0.872	0.000	−1.423	(−4.653, 1.807)	0.384	0.007
Bloodstream infection								
No	Ref				Ref			
Yes	−1.054	(−13.006, 10.898)	0.861	0.000	0.175	(−4.342, 4.693)	0.939	0.000

TMVRR, temporal muscle volume reduction rate; ATMVL, absolute temporal muscle volume loss; CI, confidence interval; GCS, Glasgow Coma Scale; ICH, intracerebral hemorrhage; NLR, neutrophil-to-lymphocyte ratio; PLR, platelet-to-lymphocyte ratio. β coefficients represent unstandardized regression coefficients from univariate linear regression. Significant associations (*p* < 0.05) are shown in bold.

### 3.2 Factors associated with temporal muscle volume change

Univariable linear regression analyses were performed to identify clinical factors associated with TMVRR and ATMVL at week 2 (3). Higher TMVRR was significantly associated with greater hemorrhage volume (*β* = 0.112, *p* = 0.001), intraventricular hemorrhage (*β* = 5.279, *p* = 0.008), greater midline shift (*β* = 0.526, *p* = 0.009), surgical treatment (*β* = 8.916, *p* < 0.001), lower Glasgow Coma Scale (GCS) score (*β* = −1.006, *p* < 0.001), pulmonary infection (*β* = 4.718, *p* = 0.020), and greater baseline TMV (*β* = 0.171, *p* = 0.027). Compared with the reference ICH Score category, TMVRR was significantly associated with an ICH Score of 1 (*β* = −1.035, *p* = 0.045), 2 (*β* = 7.682, *p* = 0.008), 3 (*β* = 7.562, *p* = 0.009), or 4 (*β* = 11.531, *p* = 0.010). Greater ATMVL was significantly associated with surgical treatment (*β* = 3.066, *p* < 0.001), lower GCS score (*β* = −0.308, *p* = 0.003), and greater baseline TMV (*β* = 0.169, *p* < 0.001). Compared with the reference ICH Score category, ICH Scores of 2 (*β* = 2.286, *p* = 0.044) and 3 (*β* = 2.225, *p* = 0.047) were also associated with greater ATMVL. Older age was associated with lower ATMVL (*β* = −0.055, *p* = 0.049). Neither TMVRR nor ATMVL was significantly associated with sex, diabetes, smoking history, alcohol abuse, intracranial infection, urinary tract infection, bloodstream infection, serum total protein, or serum albumin.

Multivariable linear regression analyses were subsequently performed to identify factors independently associated with temporal muscle loss at week 2 (Table 3). The TMVRR model had an R^2^ of 0.321 and an adjusted R^2^ of 0.233. Surgical treatment (*β* = 7.438, *p* = 0.023) and greater baseline TMV (*β* = 0.243, *p* = 0.014) were independently associated with higher TMVRR. The ATMVL model had an R^2^ of 0.555 and an adjusted R^2^ of 0.497. Surgical treatment (*β* = 3.821, *p* < 0.001), greater baseline TMV (*β* = 0.204, *p* < 0.001), and older age (*β* = 0.051, *p* = 0.048) were independently associated with greater ATMVL. Hemorrhage volume was not independently associated with either TMVRR or ATMVL after adjustment.

**Table 3:** Multivariate linear regression analysis of factors affecting the reduction in temporal muscle volume.

Variable	TMVRR (%) at Week 2			ATMVL (cm^3^) at Week 2		
	β	95% CI	*p* value	β	95% CI	*p* value
Age (years)	0.062	(−0.104, 0.229)	0.459	0.051	(0.000, 0.102)	**0.048**
Sex						
Male	Ref			Ref		
Female	2.100	(−2.952, 7.151)	0.411	0.997	(−0.548, 2.542)	0.203
Diabetes						
No	Ref			Ref		
Yes	6.068	(−1.868, 14.029)	0.132	0.966	(−1.466, 3.397)	0.432
Smoking History						
No	Ref			Ref		
Yes	−2.718	(−8.046, 2.611)	0.314	−0.945	(−2.574, 0.685)	0.253
Alcohol Abuse						
No	Ref			Ref		
Yes	2.573	(−3.902, 9.049)	0.432	0.784	(−1.196, 2.765)	0.434
Hemorrhage Volume (mL)	0.064	(−0.046, 0.174)	0.251	0.004	(−0.030, 0.038)	0.825
Baseline TMV (cm^3^)	0.243	(0.051, 0.435)	**0.014**	0.204	(0.146, 0.263)	**<0.001**
Intraventricular Hemorrhage						
No	Ref			Ref		
Yes	3.155	(−0.983, 7.294)	0.133	0.969	(−0.296, 2.235)	0.132
Midline Shift (mm)	−0.489	(−1.127, 0.149)	0.131	−0.181	(−0.376, 0.014)	0.069
GCS Score	−0.572	(−1.297, 0.152)	0.120	−0.208	(−0.429, 0.013)	0.065
Surgical Treatment						
Conservative	Ref			Ref		
Surgical	7.438	(1.069, 13.807)	**0.023**	3.821	(1.873, 5.769)	**<0.001**
Pulmonary Infection						
No	Ref			Ref		
Yes	−1.942	(−7.441, 3.557)	0.485	−1.633	(−3.315, 0.049)	0.057

TMVRR, temporal muscle volume reduction rate; ATMVL, absolute temporal muscle volume loss; CI, confidence interval. Significant associations (*p* < 0.05) are shown in bold. Model fit: TMVRR R^2^ = 0.321, adjusted R^2^ = 0.233; ATMVL R^2^ = 0.555, adjusted R^2^ = 0.497.

In a supplementary subgroup analysis, TMVRR and ATMVL at week 2 did not differ significantly between patients undergoing drainage procedures and those undergoing craniotomy-based procedures. Significant differences were observed only between surgically and conservatively managed patients (Supplementary Table S1; Supplementary Figure S1). After adjusting for age, sex, hemorrhage volume, and baseline TMV, both drainage and craniotomy-based procedures remained significantly associated with greater TMVRR and ATMVL compared with conservative management, with comparable effect sizes and widely overlapping confidence intervals between the two surgical procedures (Supplementary Table S2).

### 3.3 Muscle volume loss and functional outcome

Seven patients were excluded from the functional outcome analysis because 6-month prognostic information was unavailable, leaving 142 patients with outcome data. Patients were classified as having a favorable outcome (mRS score 0–2) or an unfavorable outcome (mRS score 3–6).

**Figure 4:**
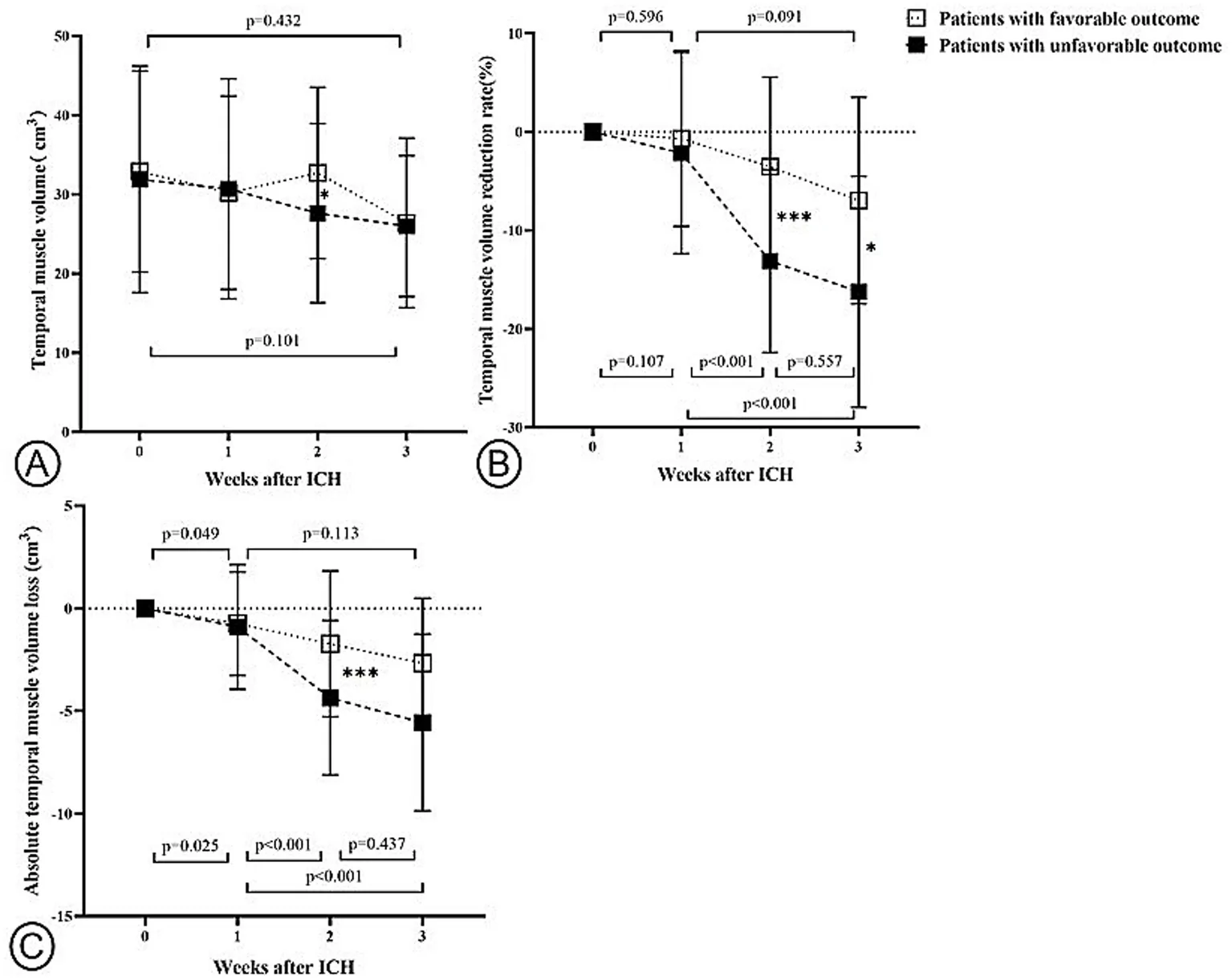
Changes in **(A)** temporal muscle volume (TMV), **(B)** temporal muscle volume reduction rate (TMVRR), and **(C)** absolute temporal muscle volume loss (ATMVL) during hospitalization are shown for the favorable-outcome group (mRS score 0–2; white squares) and the unfavorable-outcome group (mRS score 3–6; black squares). Brackets indicate pairwise comparisons between time points within each outcome group or between the two groups at the corresponding time points. Asterisks indicate statistically significant between-group differences (* *p* < 0.05, ** *p* < 0.01, *** *p* < 0.001).

At week 2, TMV was significantly lower in the unfavorable-outcome group than in the favorable-outcome group (27.622 ± 11.321 cm^3^ vs. 32.703 ± 10.820 cm^3^, *p* = 0.023; Figure 4A). No significant between-group differences in TMV were observed at the other time points.

In the unfavorable-outcome group, TMVRR and ATMVL increased significantly over time (both *p* < 0.001), with average increases of approximately 5.410 percentage points and 1.854 cm^3^ per week, respectively. In contrast, neither TMVRR nor ATMVL changed significantly over time in the favorable-outcome group (*p* = 0.091 and *p* = 0.113, respectively).

Between-group comparisons showed that TMVRR was significantly higher in the unfavorable-outcome group than in the favorable-outcome group at week 2 (13.111% ± 9.277% vs. 3.537% ± 9.093%, *p* < 0.001) and week 3 (16.231% ± 11.723% vs. 6.955% ± 10.488%, *p* = 0.031; Figure 4B). ATMVL was also significantly greater in the unfavorable-outcome group at week 2 (4.356 ± 3.759 cm^3^ vs. 1.727 ± 3.553 cm^3^, *p* < 0.001; Figure 4C). These findings indicate that patients with an unfavorable functional outcome experienced more pronounced temporal muscle loss during hospitalization.

In multivariable logistic regression analyses adjusted for age, sex, and hemorrhage volume, the associations between temporal muscle parameters and functional outcome varied according to the measurement time point (Table 4). At week 2, higher TMVRR (OR = 1.109, *p* = 0.004) and greater ATMVL (OR = 1.282, *p* = 0.009) were independently associated with an unfavorable 6-month functional outcome. Temporal muscle parameters measured at the other time points were not significantly associated with functional outcome. Admission TMV was not associated with 6-month outcome, irrespective of surgical treatment status.

**Table 4:** Logistic regression analysis of temporal muscle volume decrease and functional outcome.

Factor	OR	95% CI	*p* value
Baseline Temporal Muscle Volume(cm^3^)	1.013	(0.963,1.065)	0.624
Week 1 Temporal Muscle Volume			
TMV(cm^3^)	1.020	(0.963,1.080)	0.504
TMVRR(%)	1.031	(0.967,1.098)	0.351
ATMVL(cm^3^)	1.048	(0.846,1.299)	0.665
Week 2 Temporal Muscle Volume			
TMV(cm^3^)	0.972	(0.905,1.044)	0.540
TMVRR(%)	1.109	(1.033,1.190)	**0.004**
ATMVL(cm^3^)	1.282	(1.064,1.545)	**0.009**
Week 3 Temporal Muscle Volume			
TMV(cm^3^)	0.997	(0.867,1.146)	0.962
TMVRR(%)	1.012	(0.910,1.124)	0.830
ATMVL(cm^3^)	1.079	(0.812,1.433)	0.601

OR, odds ratio; CI, confidence interval; TMV, temporal muscle volume; TMVRR, temporal muscle volume reduction rate; ATMVL, absolute temporal muscle volume loss. Significant associations (*p* < 0.05) are shown in bold.

In univariable logistic regression analysis, higher week 2 TMVRR was associated with pulmonary infection (OR = 1.049, *p* = 0.024). However, this association was no longer statistically significant after adjustment for age, sex, and hemorrhage volume.

**Figure 5:**
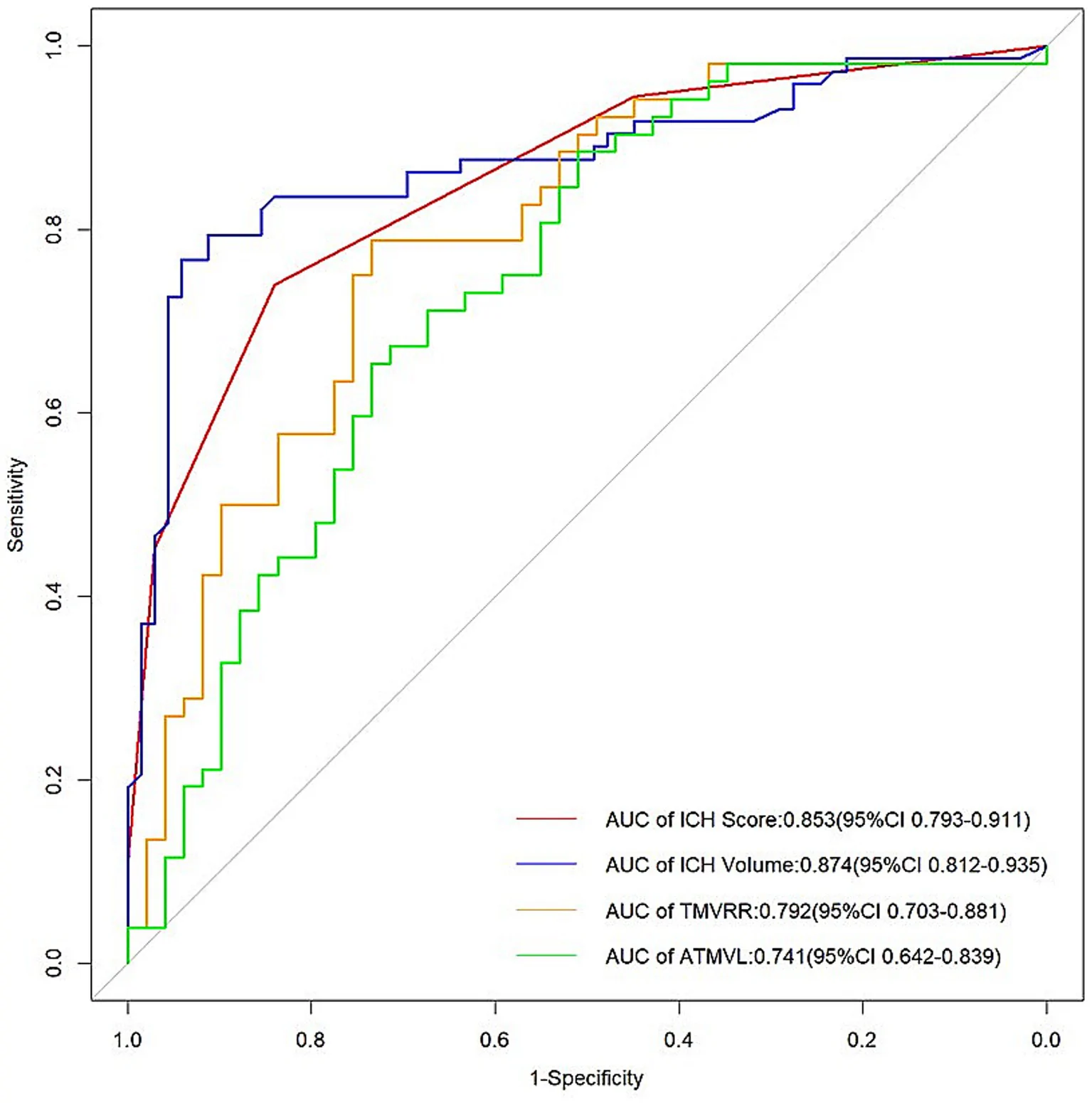
ROC curve for predicting functional outcome in HICH patients.

In the ROC analysis, hemorrhage volume yielded the largest area under the curve (AUC) for predicting an unfavorable functional outcome, followed by the ICH Score, week 2 TMVRR, and week 2 ATMVL (Figure 5). DeLong’s test showed that the only statistically significant difference was between the AUCs of hemorrhage volume and ATMVL (*p* = 0.026; Table 5). No significant differences were observed among the other predictors, supporting the potential prognostic value of week 2 TMVRR.

**Table 5:** Comparison of AUC differences in functional outcome prediction factors based on DeLong test.

Comparison Group	AUC Difference	*Z* value	*p* value
ICH Score − hemorrhage Volume	−0.021	−0.488	0.626
ICH Score − TMVRR	0.06	1.109	0.269
ICH Score − ATMVL	0.112	1.911	0.058
hemorrhage Volume − TMVRR	0.082	1.481	0.14
hemorrhage Volume − ATMVL	0.133	2.250	**0.026**
TMVRR − ATMVL	0.051	0.760	0.448

AUC, area under the receiver operating characteristic curve; TMVRR, temporal muscle volume reduction rate at week 2; ATMVL, absolute temporal muscle volume loss at week 2. A positive AUC difference indicates that the first metric performs better, while a negative value suggests the second metric is superior. Significant differences (*p* < 0.05) are shown in bold.

## 4 Discussion

This study demonstrated progressive temporal muscle loss during hospitalization in patients with HICH. Although the decline in absolute TMV across time points did not reach statistical significance, both TMVRR and ATMVL increased progressively. Temporal muscle loss was more pronounced in patients with larger hemorrhage volumes and those with unfavorable 6-month functional outcome, particularly from the second week onward. In multivariable analyses, surgical treatment and baseline TMV were independently associated with both TMVRR and ATMVL. Importantly, week 2 TMVRR and ATMVL, rather than admission TMV, were independently associated with unfavorable functional outcome after adjustment for age, sex, and hemorrhage volume.

Head CT-based temporal muscle measurements have increasingly been used as surrogate indicators of skeletal muscle status (14, 18). Kofler et al. reported an average weekly TMV loss of 7.9% in patients with spontaneous subarachnoid hemorrhage (19). In the present study, TMVRR increased by approximately 4.6 percentage points per week, indicating a comparatively lower rate of temporal muscle loss. This difference may reflect variation in disease severity, study populations, imaging schedules, and measurement methods. Kangalgil et al. reported a 15.8% reduction in rectus femoris cross-sectional area during the first week after acute brain injury (20). The greater magnitude of rectus femoris loss may be related to the larger baseline mass and greater disuse sensitivity of lower-limb muscles, as well as differences between ultrasound-based area measurements and CT-based volumetric assessment. Nevertheless, these studies consistently indicate that acute brain injury is accompanied by rapid skeletal muscle loss that can be detected across different muscle groups and imaging modalities.

Several interrelated factors may contribute to temporal muscle loss after HICH. Immobilization is likely to play an important role because patients with severe neurological deficits frequently require prolonged bed rest. Even short periods of bed rest or limb immobilization can induce substantial reductions in skeletal muscle mass and function (21). Infection may further accelerate this process. Secondary infections occur in up to approximately 30% of patients with stroke, while stroke-associated pneumonia has been reported in 8.5–14.3% of cases (22). Greater hemorrhage volume, lower GCS scores, dysphagia, endotracheal intubation, and other indicators of disease severity are also associated with an increased risk of infection (23). Persistent infection and systemic inflammation promote muscle catabolism and are associated with impaired physical function and reduced quality of life (24). In addition, mechanical ventilation and prolonged sedation may reduce muscle loading and neural activation, thereby aggravating immobilization-related protein breakdown (25). Collectively, these processes may explain why temporal muscle loss reflects not only local muscle change but also the broader systemic burden of critical illness (10, 26).

The metabolic response to critical illness may further contribute to muscle wasting. During critical illness, the body faces sustained and excessive energy demands, while glycogen reserves are rapidly depleted in the acute phase and inflammation-mediated suppression of fatty acid oxidation limits lipid-derived energy supply. Under this metabolic constraint, gluconeogenesis becomes the primary compensatory pathway for glucose homeostasis, with its requisite amino acid precursors derived almost entirely from skeletal muscle protein breakdown (27). When nutritional intake is insufficient or interrupted, net protein loss may be further amplified. A retrospective study of 1,171 ICU patients showed that the mean daily caloric intake was only 1,651 kcal/day, whereas resting energy expenditure measured by indirect calorimetry reached 1944 kcal/day, revealing a substantial energy deficit (28). Protein breakdown also increases during prolonged critical illness, contributing to a sustained negative protein balance (29). In patients with HICH, dysphagia may further restrict oral energy and protein intake (11), while clinical limitations on fluid and nutritional delivery may complicate adequate support. However, excessive or indiscriminate caloric supplementation does not necessarily prevent muscle loss and may be harmful in some critically ill populations (28). These observations suggest that muscle wasting after HICH results from the combined effects of immobilization, inflammation, metabolic stress, and inadequate nutritional intake.

Surgical treatment, rather than hemorrhage volume, remained independently associated with TMV loss in the multivariable models. This finding should not be interpreted as evidence that surgery directly causes muscle wasting. Surgical intervention is generally reserved for patients with more severe clinical presentations and may therefore capture several dimensions of disease severity, including impaired consciousness, mass effect, neurological deterioration, and the need for prolonged critical care. The disappearance of the independent association between hemorrhage volume and TMV loss after adjustment may reflect overlap between hemorrhage burden, surgical treatment, and other severity-related variables. Moreover, the supplementary analysis showed no significant difference in week 2 TMVRR between drainage procedures and craniotomy-based surgery, although the limited subgroup sizes reduced the power of this comparison.

Greater baseline TMV was independently associated with both TMVRR and ATMVL. Patients with greater initial muscle volume may have more absolute muscle tissue available for loss, and regression toward the mean may also contribute to this association. Older age was independently associated with greater ATMVL after adjustment, although the corresponding association with TMVRR was not significant. Because the direction of the age association differed between the univariable and multivariable analyses, this result may be influenced by confounding or interactions with baseline muscle volume and should be interpreted cautiously.

A central finding of this study was that dynamic TMV changes at week 2 showed greater prognostic relevance than TMV measured at admission. Previous studies examining admission temporal muscle measurements have produced inconsistent results. Pesonen et al. reported that patients undergoing intracerebral hemorrhage evacuation with low total temporal muscle thickness (TMT) exhibited shorter overall survival and poorer functional outcome compared to those with normal muscle status (30). In contrast, Nozoe et al. found that disease severity and comorbidity, rather than TMT, independently predicted functional outcome in older patients with ICH (31). Gubarev et al. similarly reported that TMT was no longer independently associated with outcome after adjustment for age and hemorrhage volume (32). Consistent with the latter findings, admission TMV was not associated with 6-month functional outcome in the present cohort, regardless of surgical treatment status.

Baseline TMV primarily reflects premorbid muscle reserve and is strongly influenced by age, sex, body size, and nutritional status (33). A single admission measurement therefore cannot capture the cumulative effects of immobilization, infection, nutritional insufficiency, and sustained catabolism during hospitalization. By evaluating changes relative to baseline, TMVRR may reduce the influence of between-patient variation and provide a more direct measure of acute muscle deterioration. Changes observed during the first week may have been too small to provide stable prognostic information. By week 2, however, the accumulated effects of critical illness were more apparent, and both TMVRR and ATMVL were independently associated with functional outcome. These findings identify the second week of hospitalization as a potentially informative time window for assessing clinically meaningful muscle loss.

Acute muscle wasting may contribute to weakness, impaired mobility, prolonged mechanical ventilation, and delayed rehabilitation. Persistent disability after critical illness is also associated with impaired muscle regeneration, mitochondrial dysfunction, fibrosis, inflammation, and reduced muscle quality (34). In a long-term follow-up study of survivors of acute respiratory distress syndrome, pulmonary function largely recovered, whereas exercise capacity and physical quality of life remained impaired 5 years later, suggesting that systemic weakness may persist beyond recovery from the primary organ injury (35). Substantial muscle loss commonly occurs during the first week of intensive care, and many survivors experience prolonged weakness and limitations in daily activities (36). These persistent impairments reflect interacting neuroendocrine, vascular, neurological, inflammatory, mitochondrial, and regenerative abnormalities (37).

In patients with stroke, muscle loss may compound the disability directly caused by neurological injury. Hemiparesis, impaired coordination, dysphagia, and reduced activity limit rehabilitation from the onset, while systemic muscle depletion further reduces the physical reserve required for recovery. Gwak et al. reported that dysphagia, early neurological deterioration, and limited post-discharge recovery partially mediated the association between temporal muscle-defined sarcopenia and poor 3-month outcome in older patients with acute stroke (38). A scoping review similarly concluded that stroke-related sarcopenia is associated with impaired motor, swallowing, and neurological function, as well as increased recurrence and mortality risks (39). Thus, temporal muscle loss may reflect a clinically relevant component of the interaction between primary neurological injury and secondary systemic deconditioning. Nevertheless, the observational design of the present study does not establish that temporal muscle loss directly causes unfavorable outcome.

In the ROC analysis, TMVRR yielded a larger AUC than ATMVL, although the difference was not statistically significant. Relative change may provide greater discrimination because it accounts for the substantial interindividual variation in baseline temporal muscle size. However, hemorrhage volume remained the strongest individual predictor, and only the difference between hemorrhage volume and ATMVL reached statistical significance. Accordingly, TMV-related parameters should be regarded as complementary rather than replacement prognostic indicators.

Several interventions have been proposed to reduce muscle wasting in critically ill patients, including early mobilization, minimization of sedation, appropriate protein delivery, glycemic management, and neuromuscular electrical stimulation (40). Serial TMV assessment may offer a convenient means of identifying patients with rapid muscle loss because it can be incorporated into clinically indicated head CT examinations without additional imaging or patient participation. A marked decline in TMV may prompt closer evaluation of nutritional adequacy, infection, immobilization, and rehabilitation needs. Nevertheless, the present findings do not establish that TMV-guided interventions improve outcome, and this potential application requires prospective validation.

This study has several limitations. First, its single-center retrospective design introduces the possibility of selection bias. Patients with mild HICH generally undergo fewer repeated CT examinations and may therefore have been underrepresented, whereas patients with more severe disease may have contributed more follow-up scans. Second, nutritional data, including energy and protein intake, were unavailable, preventing assessment of their relationships with TMV loss. Third, only thalamic and basal ganglia hemorrhages were included; whether the findings apply to lobar, brainstem, or cerebellar hemorrhage remains uncertain. Fourth, the limited sizes of the surgical subgroups precluded reliable comparisons among minimally invasive drainage, neuroendoscopic surgery, microsurgical evacuation, and decompressive craniectomy. Fifth, CT examinations were obtained within predefined intervals rather than at identical time points. The number of available scans also declined substantially from admission to week 3, and the available-case analysis may have preferentially included patients with more severe or prolonged clinical courses. Sixth, 7 of the 149 enrolled patients (4.7%) were lost to follow-up and were excluded from the prognostic analyses. It is not known whether the patients lost to follow-up were comparable to those included in the analyses. Finally, the relatively short enrollment period and modest sample size limited the precision of subgroup and multivariable analyses. Prospective multicenter studies with standardized imaging time points, detailed nutritional and rehabilitation data, repeated outcome assessments, and predefined TMV measurement protocols are needed to validate these findings.

## 5 Conclusion

Temporal muscle loss progresses during hospitalization in patients with HICH and is more pronounced among those with severe clinical presentations and unfavorable functional outcome. Week 2 TMVRR and ATMVL, rather than admission TMV, were independently associated with 6-month functional outcome. Serial TMV assessment using routine head CT may provide a practical, noninvasive marker of acute muscle loss and may complement established indicators of HICH severity and prognosis. Prospective studies are required to determine its clinical utility and whether TMV-guided supportive interventions can improve functional recovery.

## Data Availability

The raw data supporting the conclusions of this article will be made available by the authors, without undue reservation.
