# Supplementary material for: Predictive value of changes in temporal muscle volume for 6-month functional outcome in patients with hypertensive intracerebral hemorrhage
Source: Front Neurol. 2026 Aug 12;17:1751620. doi: 10.3389/fneur.2026.1751620 (PMC13506393; doi:10.3389/fneur.2026.1751620)
Supplement: Supplementary file 1 [file Image_1.pdf]

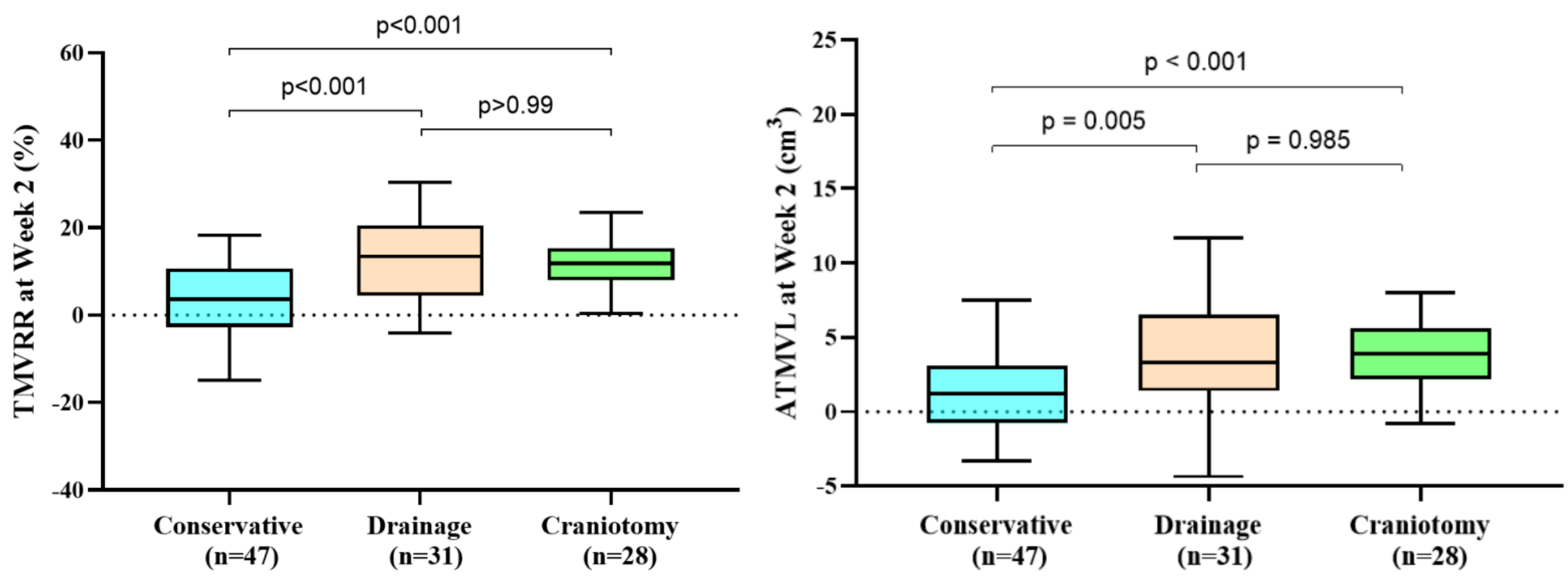

Supplementary Figure 1. Temporal muscle volume reduction at week 2 by surgical procedures. TMVRR: temporal muscle volume reduction rate; ATMVL: absolute temporal muscle volume loss. Brackets indicate pairwise comparisons among groups, with p values labeled above each bracket.
