# Supplementary material for: Predictive value of changes in temporal muscle volume for 6-month functional outcome in patients with hypertensive intracerebral hemorrhage
Source: Front Neurol. 2026 Aug 12;17:1751620. doi: 10.3389/fneur.2026.1751620 (PMC13506393; doi:10.3389/fneur.2026.1751620)
Supplement: Supplementary file 2 [file Table_1.docx]

Supplementary Table 1 Descriptive Statistics of Temporal Muscle Volume Reduction at Week 2 by Treatment Procedures

| Group | Procedures | n | TMVRR (%), mean ± SD | ATMVL (cm³), mean ± SD |
| --- | --- | --- | --- | --- |
| Conservative | — | 47 | 3.37 ± 8.36 | 1.35 ± 2.71 |
| Drainage group | EVD or hematoma puncture drainage | 31 | 11.91 ± 10.75 | 4.57 ± 4.82 |
| Craniotomy group | Hematoma evacuation or decompressive craniectomy | 28 | 12.70 ± 9.12 | 4.24 ± 3.29 |
| TMVRR: temporal muscle volume reduction rate; ATMVL: absolute temporal muscle volume loss; EVD: external ventricular drainage; SD: standard deviation. | | | | |
