# Supplementary material for: Predictive value of changes in temporal muscle volume for 6-month functional outcome in patients with hypertensive intracerebral hemorrhage
Source: Front Neurol. 2026 Aug 12;17:1751620. doi: 10.3389/fneur.2026.1751620 (PMC13506393; doi:10.3389/fneur.2026.1751620)
Supplement: Supplementary file 3 [file Table_2.docx]

Supplementary Table 2 Linear Regression Analysis of Surgical Procedures and Temporal Muscle Volume Reduction

| Variable | TMVRR (%) at Week 2 | | ATMVL (cm³) at Week 2 | |
| --- | --- | --- | --- | --- |
|  | β (95% CI) | P value | β (95% CI) | P value |
| **Surgical Procedure** |  |  |  |  |
| Conservative | Ref |  | Ref |  |
| Drainage group | 7.474 (2.627, 12.321) | **0.003** | 3.077 (1.574, 4.579) | **<0.001** |
| Craniotomy group | 8.823 (2.694, 14.952) | **0.005** | 3.508 (1.608, 5.408) | **<0.001** |
| TMVRR: temporal muscle volume reduction rate; ATMVL: absolute temporal muscle volume loss; CI: confidence interval. Significant associations (p < 0.05) are shown in bold. Model fit: TMVRR: R² = 0.258, adjusted R² = 0.213; ATMVL: R² = 0.501, adjusted R² = 0.470. | | | | |
